# LSPR-mediated high axial-resolution fluorescence imaging on a silver nanoparticle sheet

**DOI:** 10.1371/journal.pone.0189708

**Published:** 2017-12-15

**Authors:** Eiji Usukura, Yuhki Yanase, Ayumi Ishijima, Thasaneeya Kuboki, Satoru Kidoaki, Koichi Okamoto, Kaoru Tamada

**Affiliations:** 1 Institute for Materials Chemistry and Engineering, Kyushu University, Fukuoka, Japan; 2 Graduate School of Science, Nagoya University, Nagoya, Japan; 3 Graduate School of Biomedical & Health Science, Hiroshima University, Hiroshima, Japan; Pennsylvania State Hershey College of Medicine, UNITED STATES

## Abstract

This paper reports our original technique for visualizing cell-attached nanointerfaces with *extremely high axial resolution* using homogeneously excited localized surface plasmon resonance (LSPR) on self-assembled silver nanoparticle sheets. The LSPR sheet can confine and enhance the fluorescence at the nanointerface, which provides high signal-to-noise ratio images of focal adhesion at the cell-attached interface. The advantage of this LSPR-assisted technique is its usability, which provides comparable or higher-quality nanointerfacial images than TIRF microscopy, even under epifluorescence microscopy. We also report the cytotoxicity of silver nanoparticles, as determined via morphological analysis of adherent cells on the sheet.

## Introduction

Recently, the demand for super-resolution fluorescence microscopy is increasing within the field of cell biology because of the requirement to investigate molecular-level dynamic reactions in or near cells[1–3]. Not only is confocal laser scanning microscopy (CLSM) utilized as a standard tool for high-resolution fluorescence imaging, simulated emission depletion (STED) microscopy, structured illumination microscopy (SIM), photoactivated localization microscopy (PALM), and stochastic optical reconstruction microscopy (STORM) are also utilized as a family of super-resolution microscopy techniques. These super-resolution microscopy techniques have an advantage in their lateral resolution but are not as advantageous in either their axial resolution or temporal resolution because of their scanning criteria[4]. Hence, these techniques are not adequate for imaging rapid dynamics in a living system. Total internal reflection fluorescence (TIRF) microscopy is known to provide the highest axial and temporal resolution compared with the other super-resolution microscope systems[5]. TIRF microscopy is suitable for real-time imaging of cell/substrate contact regions (100–200 nm region from the top surface of a cover slip) where cellular dynamics can be observed. The problem with this state-of-the-art technology is the cost of the apparatus, which prevents it from being standard equipment in basic laboratories.

In our previous study, we proposed a simple, effective method for visualizing the nanointerface of adhesive cells using localized surface plasmon resonance (LSPR) excited on a two-dimensional (2D) self-assembled gold nanoparticle (NP) sheet[6]. The light confined by LSPR detects fluorescence in a region of only a few tens of nanometres at the interface, which creates notably high ‘axially’ confined imaging that is superior to any other super-resolution microscopic techniques, including TIRF microscopy[5]. In general, surface plasmon-related imaging technique utilizes metal-thin-film-mediated ‘propagating’ SPR, which enhances the fluorescence in a 100–200 nm region from the metal surface[7]. However, the propagating property often deteriorates the spatial resolution of the images. Unlike the ‘propagating’ SPR-mediated imaging, our LSPR technique exhibits extremely high axial confinement and practically high lateral resolution because there is little overlap of the excited fluorescence molecules in the depth direction[6]. In this paper, we utilized a silver nanoparticle sheet for this application[8, 9]. The silver nanoparticles are expected to exhibit a stronger LSPR field than the gold nanoparticles. However, there are several potential risks, e.g., stability of the silver nanoparticles in aqueous solution (oxidation) and cytotoxicity (biocompatibility)[10, 11]. Here, we report the cytotoxicity of silver nanoparticles to adhesive cells by using a homogeneous self-assembled silver nanoparticle sheet as the test surface, in parallel to the quality test of the LSPR-mediated fluorescence imaging.

## Material and methods

### Silver nanoparticles sheet

Silver nanoparticles capped with myristates (AgMy; *d* = 5 nm) were synthesized by thermolysis as described in a previous report [8, 12]. The yellow-coloured homogeneous AgMy sheet (monolayer) was fabricated by self-assembly of the particles at the air-water interface. The sheet was transferred onto hexamethyldisilazane (HMDS)-treated hydrophobized cover glass using the Langmuir-Schaefer method at a surface pressure (Π) of 15 mN/m. The scanning electron microscope (SEM) image revealed that the interparticle distance of the hexagonally packed AgMy was approximately 2.0 nm. The AgMy sheet exhibits the LSPR peak at 480 nm[8].

### Rat basophilic leukaemia cells

Rat basophilic leukaemia (RBL-2H3) cells were cultured in Roswell Park Memorial Institute (RPMI) 1640 medium supplemented with 10% foetal calf serum (FCS), 100 U/mL penicillin, and 100 μg/mL streptomycin [6]. The day before the experiment, RBL-2H3 cells were harvested using trypsin. A flexiPERM^®^ chamber conA (φ12 mm, Greiner Bio One) was placed on the glass slip half-covered with the AgMy sheet. The substrates were sterilized by UV irradiation for 30 min prior to use. The RBL-2H3 cells in RPMI 1640 medium were then placed into the well and cultured for the indicated time (1 or 2 hours or overnight) in a CO_2_ incubator (4% CO_2_ concentration) at 37°C. The detailed procedure was described in our previous paper [6].

### NIH-3T3 fibroblast cells

NIH-3T3 fibroblasts with stably expressed venus-paxillin were also utilized to examine the cytotoxicity of silver nanoparticles and the quality of LSPR-mediated fluorescence images. The detailed procedure for cell culture and fluorescent labeling were described in our previous paper [6]. In this study, we fixed the cells by paraformaldehyde before imaging.

## Results and discussion

**Fig 1:**
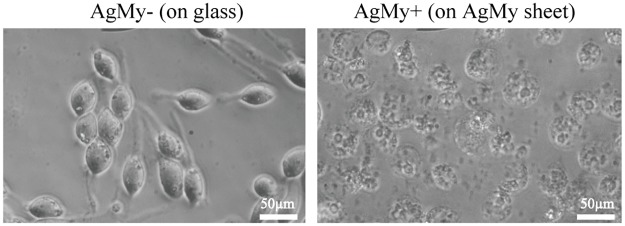
Phase contrast optical microscopic images of the cells attached on glass (AgMy-) and on the AgMy sheet (AgMy+). These cells were cultured overnight.

**Fig 2:**
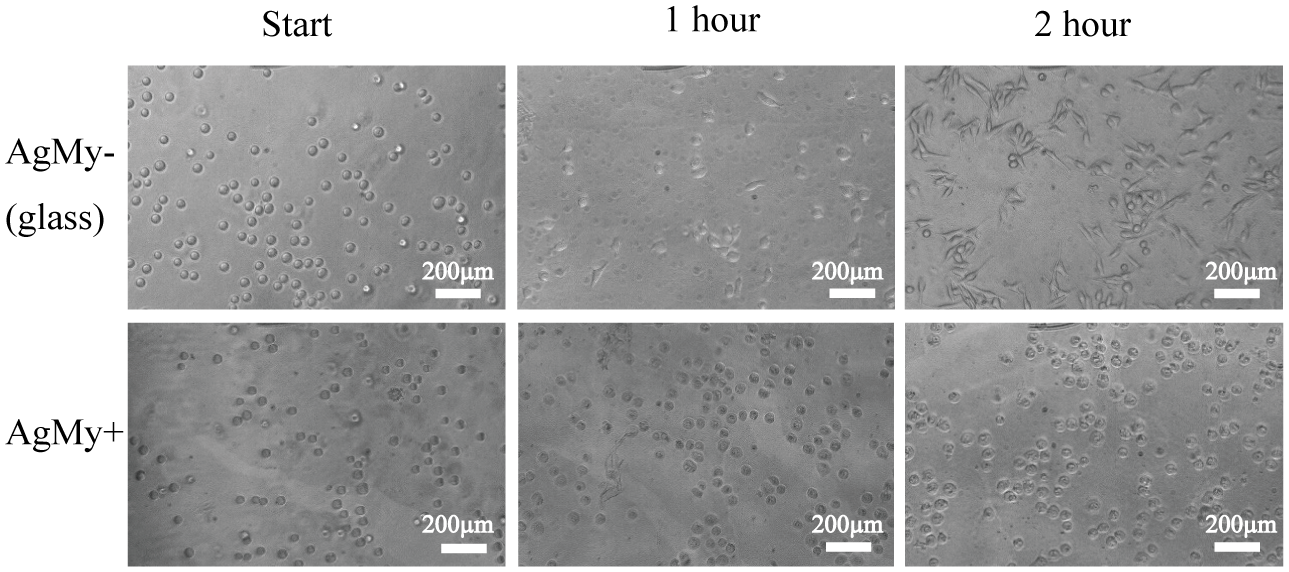
Cell-cultured surface immediately after seeding and 1 or 2 hours later on glass (AgMy-) and on the AgMy sheet (AgMy+).

Fig 1 shows typical phase contrast optical microscopic images of RBL-2H3 cells attached to glass (AgMy-) and to the AgMy sheet (AgMy+) after overnight incubation. The cells exhibited elongated shapes on the glass, which corresponds to the morphology of active cells, as described in detail previously [6]. Conversely, the cells on the AgMy sheet exhibited shrinkage and fragmentation, which is the typical morphology of apoptotic cells. Since the cells were cultured in the same chamber, cytotoxicity was not caused by dissolving the silver ions in the solution. Fig 2 shows the timed observations of the cell morphology immediately after cell attachment and 1 and 2 hours later. Initially, the cells were round on both the glass and the AgMy sheet. However, they began to elongate after a few hours of adhesion on the glass substrate. In contrast, the cells retained their round shape on the AgMy sheet. Fragmentation and aggregation of intracellular materials (a sign of apoptosis) was not apparent in the first hour of adhesion but began to show after 2 hours. The optical microscopic images of the RBL-2H3 cells attached to oleylamine-capped gold nanoparticles (AuOA) after overnight incubation is shown for comparison in the supporting data. The cells on the AuOA sheet exhibited comparable morphology to those on the glass as reported in our previous study[6]. Thus, the difference in cytotoxicity between the gold and silver nanoparticles was clear in the cell-adhesion test on the self-assembled 2D sheet.

**Fig 3:**
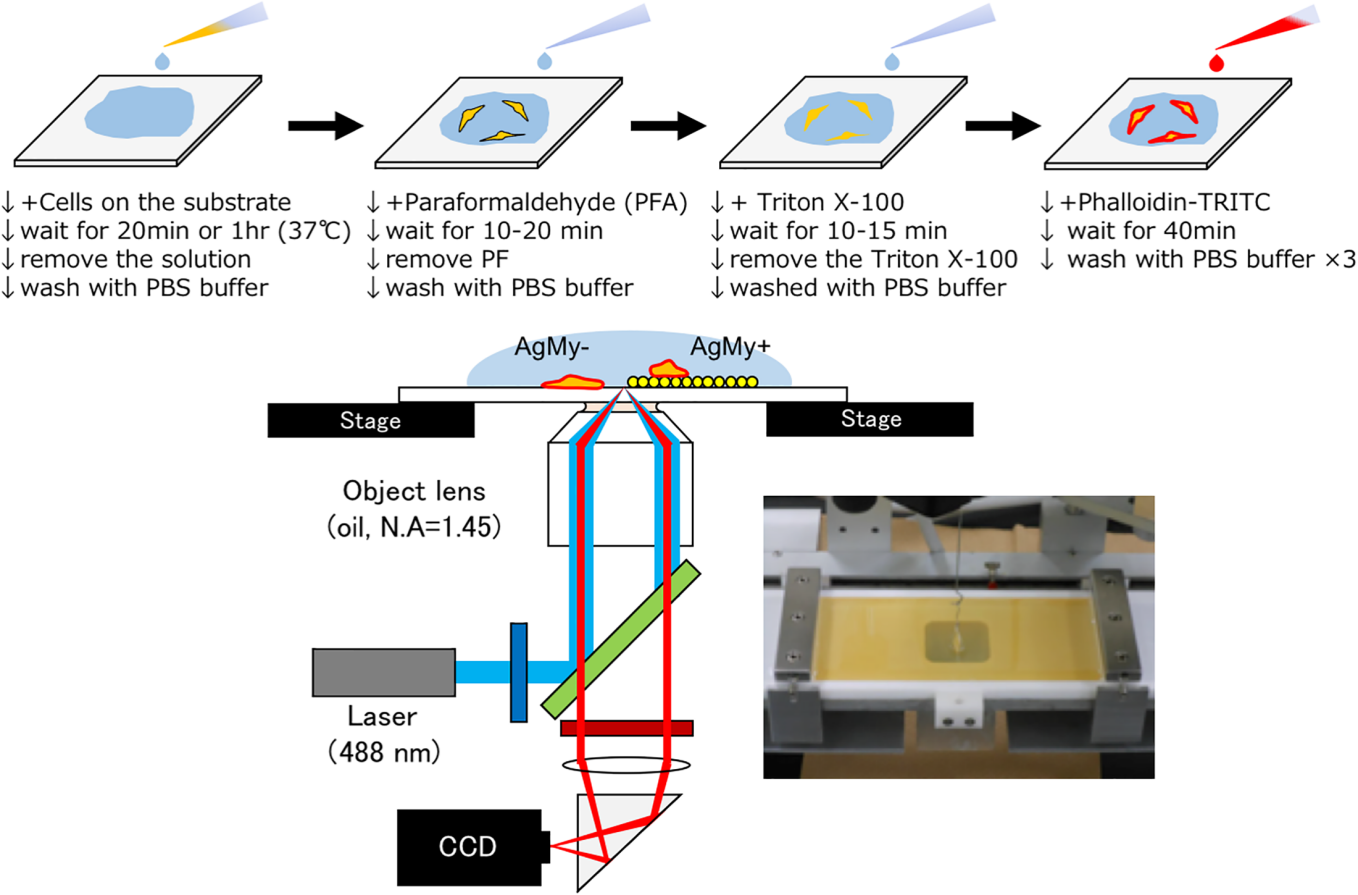
Cell culture and fluorescence labelling procedure (above) and total internal reflection fluorescence (TIRF) microscope system on glass (AgMy-) and the AgMy sheet (AgMy+)(bottom).

For the fluorescence imaging on the AgMy sheet, we selected a “short” incubation time, either 20 min or 60 min, to complete the cell imaging before the cell death. Fig 3 shows the cell culture procedure, immobilization and fluorescence labelling for the TIRF imaging. First, adherent cells were fixed with paraformaldehyde (PFA) and treated with Triton X-100. Then, the actin filaments in the cells were stained with fluorescence dyes by adding TRITC-conjugated Phalloidin (Sigma-Aldrich) as reported in our previous study[6]. After staining, a clean cover glass was superimposed onto the cell-attached cover glass and fixed with double-faced tape between them. The TIRF imaging was conducted using a customized TIRF microscope (ECLIPSE Ti, Nikon, Japan) equipped with a multiple-wavelength laser (LightHUB, Omicron-Laserage, Laserprodukte GmbH, Germany), a high-speed CCD camera (EM-CCD C9100-13, Hamamatsu Photonics, Japan) and a 100× objective lens (CFI Apo TIRF 100×H/1.49, Nikon, Japan)[6, 8]. The multiple-wavelength laser was used to choose the optimum incident wavelength in consideration of the overlap with the LSPR wavelength of the AgMy sheet and the excitation/emission wavelengths of the fluorescent dyes. In this study, 488 nm lasers (5 mW) and 525 emission filters were applied for imaging (see supporting data). The incidence angle was adjusted from perpendicular to beyond the total internal reflection angles. The images were captured with a 30.5 msec/frame exposure time. All images were collected without an aperture stop (AS) and shown without contrast control via graphics processing software, in the same manner as our previous study[6].

**Fig 4:**
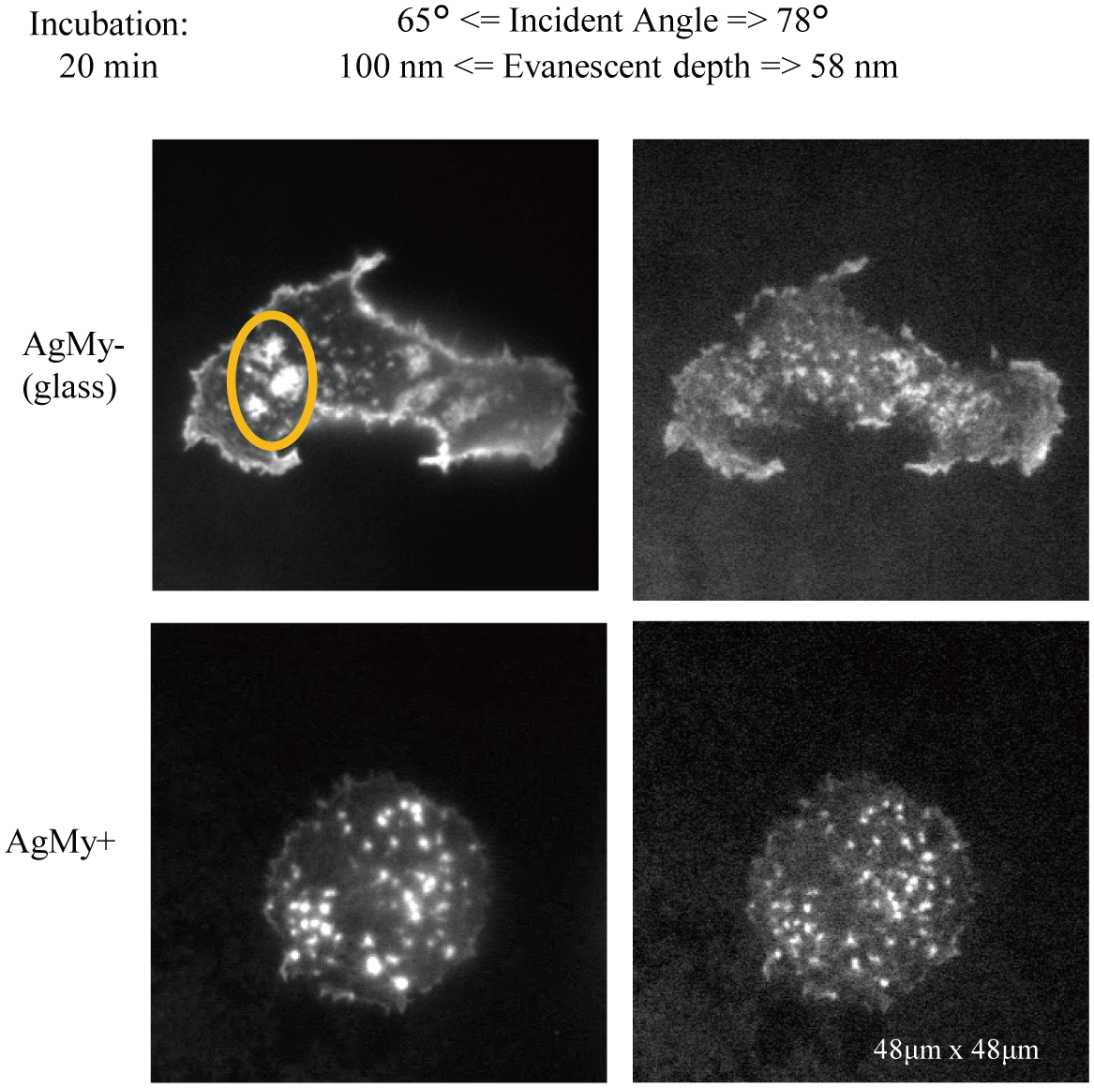
Comparison of fluorescence images of TRITC-labelled actin filaments in RBL-2H3 cells on glass (AgMy-) and on the AgMy sheet (AgMy+) in aqueous media. The incubation time for the cell adhesion was 20 min. The incident light was 488 nm in wavelength, and the intensity was 5 mW. The incidence angle was 65° (left) and 78° (right) when the critical TIR angle was 61.4°.

Fig 4 shows the TIRF fluorescence images of the Phalloidin-TRITC-labelled actin filaments in the RBL-2H3 cells with an incubation time of 20 min on the glass (AgMy-) and on the AgMy sheet (AgMy+). The images were taken at two different incidence angles, 65° and 78° in water, which correspond to the evanescent depths of 58 nm and 100 nm, respectively. On the glass substrate (AgMy-), especially at the 65° incidence angle, the dot-like emission from the bottom of the cells (focal adhesion) and the emission from the periphery of the cells (near the cell membrane) were observed together. When the incidence angle increased to 78°, the emission from the periphery of the cells was weakened, and only the emission from the focal adhesion was observed. These results are consistent with the expectation for the evanescent depth[13]. When the evanescent depth was large (~100 nm), the emission from the periphery was integrated to the vertical direction and appeared to be strong. When the evanescent depth was small (~58 nm), the emission from the bottom of the cells remained unchanged since the emitting position was less than 58 nm from the interface, while the emission from the periphery was weakened since the dyes that were more than 58 nm from the interface were not excited. The brightest three spots are marked by an orange circle, which disappears from the image at the 78° incidence angle and must be located at a distance of more than 58 nm as well. Due to the effect of evanescent depth, the image taken at the larger incidence angle revealed a more detailed structure at the cell-attached interface. In contrast, the images on the AgMy sheet (AgMy+) do not change with the incidence angles. In both images at the 65° and 78° incidence angles, only focal adhesions were visualized (no emission from the periphery). These results revealed that the emission occurred only near the interface (less than 58 nm), as expected from the theoretical calculation. In our previous study, we reported that the optical field intensity excited by LSPR on the AgMy sheet was confined to less than a 10 nm region from the centre of the particles, based on the finite-difference time-domain (FDTD) calculation[8]. The calculated confined distance should be slightly shorter than the actual values, as discussed in our latest publication on the gold nanoparticle sheet, due to the effect of long-distance plasmon coupling on the 2D sheet[6]. However, the obtained images were consistent with the theoretical expectation.

**Fig 5:**
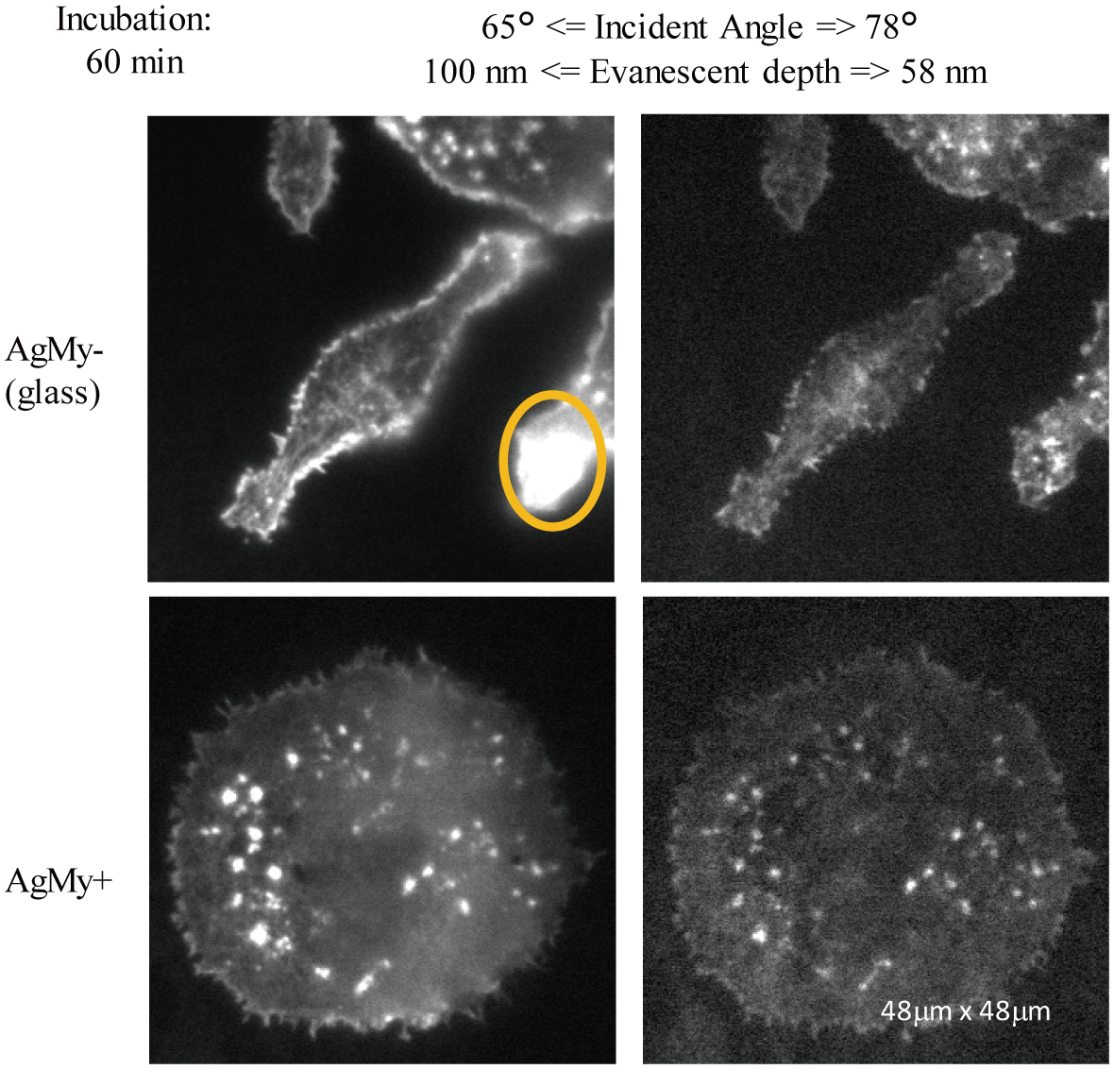
Comparison of fluorescence images of TRITC-labelled actin filaments in RBL-2H3 cells on glass (AgMy-) and on the AgMy sheet (AgMy+) in aqueous media. The incubation time for the cell adhesion was 60 min. The imaging condition was the same as Fig 4.

Fig 5 shows the TIRF fluorescence images of the same cells with an incubation time of 60 min. These images were also taken at the 65° and 78° incidence angles. Here, the cells on the glass (AgMy-) exhibited a largely elongated structure, while those on the AgMy sheet (AgMy+) did not show a morphology change and remained round. The periphery of the cells on the glass was brighter on the glass at the smaller 65° incidence angle, with good reproducibility. The brightest spot (right side, marked by an orange line) disappeared together with the periphery at the 78° incidence angle, with good reproducibility as well. The images on the AgMy sheet do not change with the incidence angles in the same way they do in Fig 4 because of the emission from the confined nanointerface.

**Fig 6:**
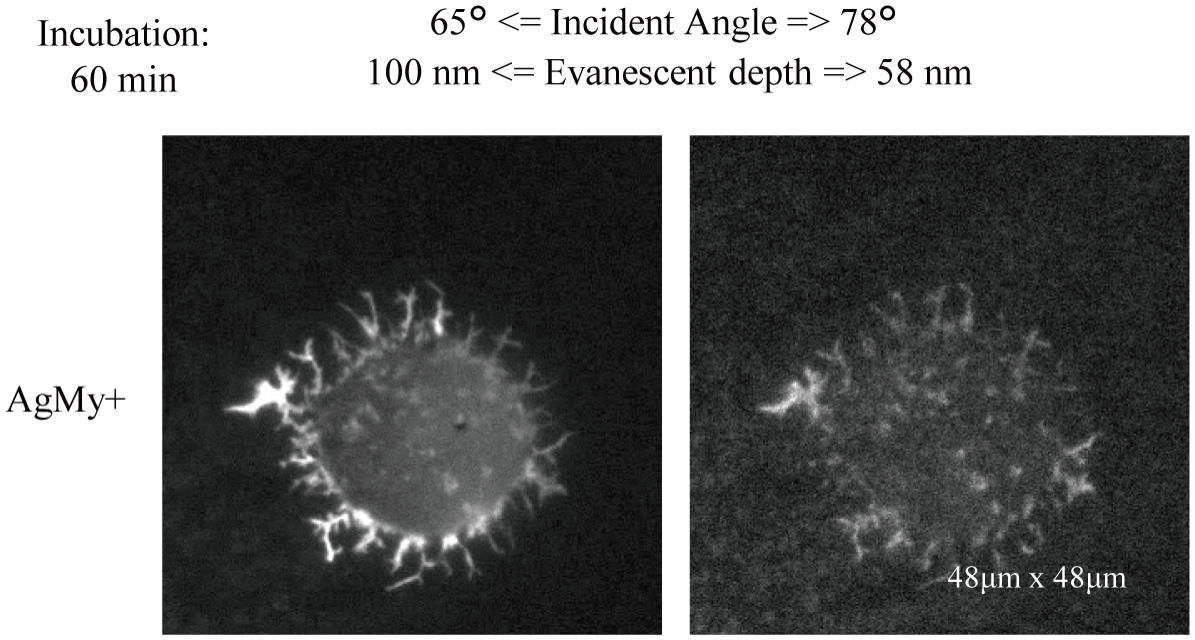
Image of TRITC-labelled actin filaments in RBL-2H3 cells on the AgMy sheet (AgMy+) in aqueous media. The incubation time for the cell adhesion was 60 min. The “pickle-like” morphology of the cells revealed that the cells are under stress due to contact with the AgMy sheet.

The quality of the nanointerfacial image taken on the AgMy sheet was as good as that taken on the gold nanoparticles[6]. The only difference is the cytotoxicity. The cells exhibited an elongated shape on the gold nanoparticle sheet at both 20 min and 60 min, as well as during overnight incubation times. On the other hand, the cells on the AgMy sheet did not reach apoptosis after a few hours but were found in an “unhealthy” condition. The cells remained round, as shown in Figs 4 and 5, and some of the cells held a “pickle-like” structure composed of labelled actin filaments, which is the typical morphology of RBL-2H3 cells under stress (Fig 6). This structure was clearly visualized in our LSPR-mediated nanointerfacial image.

**Fig 7:**
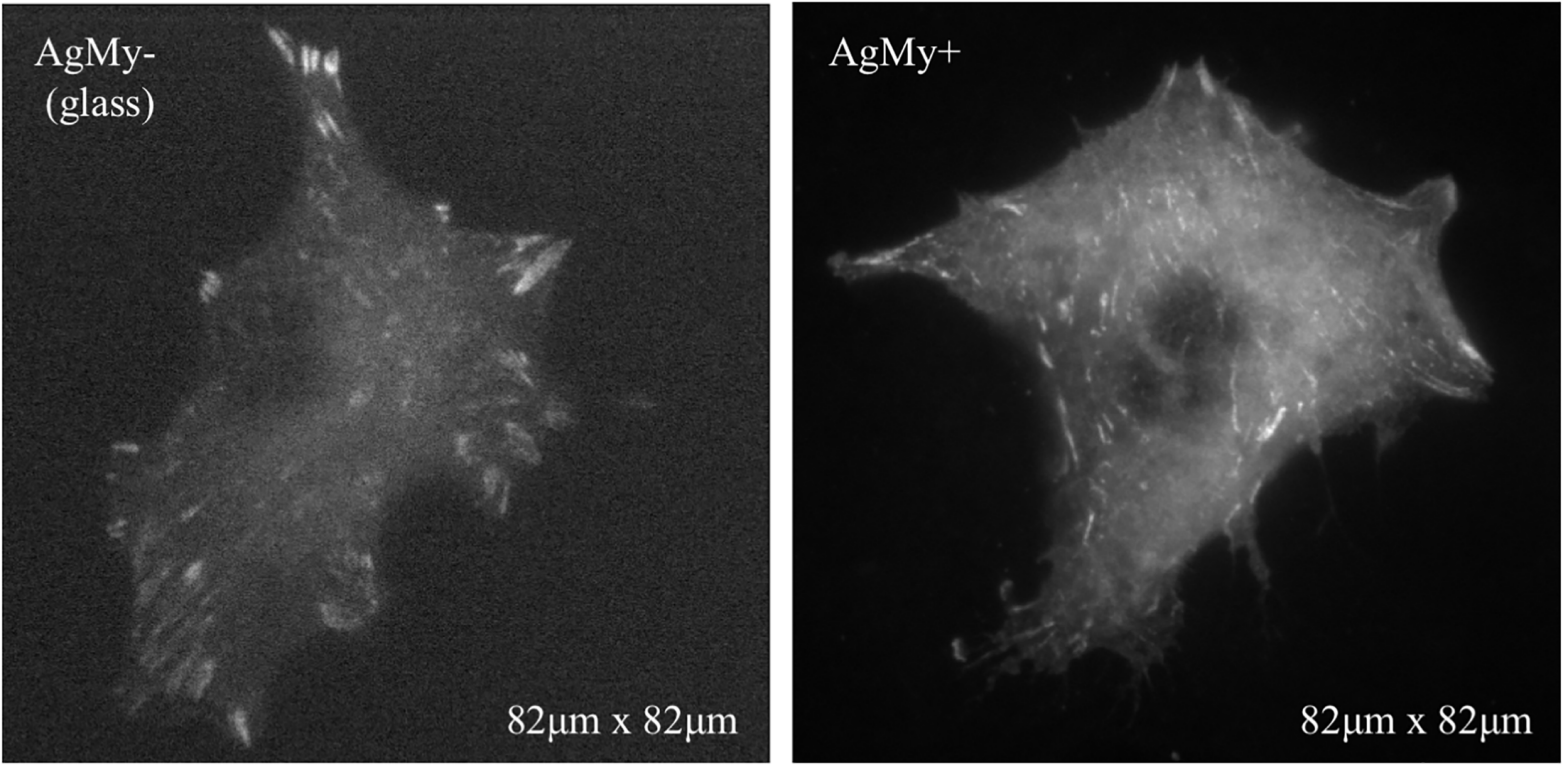
Images of venus-paxillin-expressing NIH-3T3 cells on glass (AgMy-) and on the AgMy sheet (AgMy+) taken by a super-resolution digital CMOS camera (65 nm/pixel).

All the data taken on the AgMy sheet revealed a clear focal adhesion, which clearly shows the efficiency of the self-assembled metal nanoparticle sheet as a substrate for nanointerfacial imaging, in the same manner as on AuOA sheet. Although silver nanoparticles raise concern due to their possible cytotoxicity, they are still a necessary component of the self-assembled nanoparticle sheets for this application, since they cover a different LSPR wavelength region than the gold nanoparticles and accommodate different dyes (blue to green emissions)[14]. As a matter of fact, when different cells were utilized for imaging, the cytotoxicity problem was less critical. Fig 7 shows an image of venus-paxillin-expressing NIH-3T3 cells (λex 520 nm; λem 540 nm) fixed by paraformaldehyde on the AgMy sheet under a TIRF microscope. The image was taken by a laser with a wavelength of 488 nm (7 mW), and the exposure time was 30.5 msec. Here we used a super-resolution digital CMOS camera (65 nm/pixel, ORCA-Flash 4.0, Hamamatsu, Japan). The image on AgMy sheet exhibited the morphology quite similar to that on glass, and revealed quite detailed, high quality of fluorescence image. A cell body appeared slightly round and brighter on AgMy sheet compared with that on glass.

Although no critical cytotoxic reaction was observed for this case, the vacuum deposition of the SiO_2_ layer can be used if the cytotoxicity of AgMy sheet is not negligible or an oxidation of AgMy sheet is unavoidable in culture media. Some of the proteins can be used for a biocompatible spacer layer as well (see supplementary data), although nano-order thickness control needs to be done[15].

## Conclusions

We have demonstrated the fluorescence imaging of the cell-attached nanointerface using a self-assembled silver-NP sheet (AgMy). We proved the high potential of the metallic nanoparticle sheet for use as a fluorescence imaging substrate (enhanced fluorescence with axial confinement of light for non-scanning, high-speed imaging) for silver nanoparticles in a similar manner to gold nanoparticles. This technique provides the opportunity for high-resolution imaging even under conventional epifluorescence microscopy^6)^, which will open the possibility for all biochemists and medical scientists to perform state-of-the-art molecular imaging using their own microscope.

## Supporting information

S1 Fig Optical microscope images of cells attached on glass and the AuOA sheet. These cells were cultured independently in isolated chambers overnight.
(TIF)

S2 Fig LSPR spectrum of AgMy sheet and Ex/Em spectra of TRITC dye. (TIF)

S3 Fig Comparison of fluorescence images of Alexa-Fluor 647-labeled actin filaments in RBL-2H3 cells on AuOA+ in aqueous media. The incubation time for the cell adhesion was 20 min and 60 min. The incubation time for the cell adhesion was 20 min. The incident light was 591 nm in wavelength, and the intensity was 5 mW. The incident angle was 65° (left) and 78° (right). The exposure time was 500 msec.
(TIF)

S4 Fig Images of venus-paxillin-expressing NIH-3T3 cells fixed by paraformaldehyde on glass, on AgMy sheet, and on AgMy sheets covered with 5 nm and 10 nm SiO_2_ layers. The images were taken by a super-resolution digital CMOS camera (65 nm/pixel). The incident light was 488 nm in wavelength, and the intensity was 7 mW. The incident angle was 62°. The exposure time was 300 msec.
(TIF)

S5 Fig Improved RBL-2H3 cell adhesion by adding collagen or fibronectin on top of AgMy sheet. The collagen or fibronectin were spin-coated on top of AgMy sheet. On fibronectin, some cells showed still apoptotic morphology (see the magnified image), which may be due to the inhomogeneous coating with fibronectin in spin-coating.
(TIF)
