# Supplementary figures and images for: LSPR-mediated high axial-resolution fluorescence imaging on a silver nanoparticle sheet
Source: PLoS One. 2017 Dec 15;12(12):e0189708. doi: 10.1371/journal.pone.0189708 (PMC5731743; doi:10.1371/journal.pone.0189708)

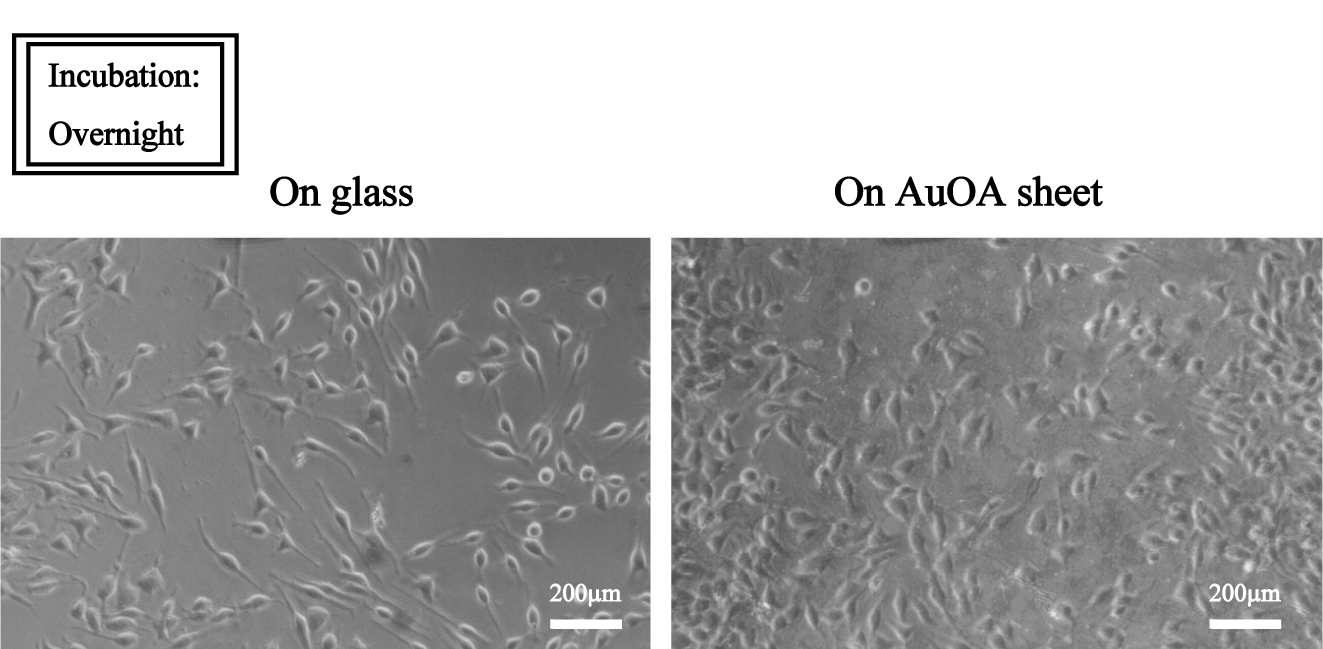

Supplement: S1 Fig — These cells were cultured independently in isolated chambers overnight. (TIF) [file pone.0189708.s001.tif]

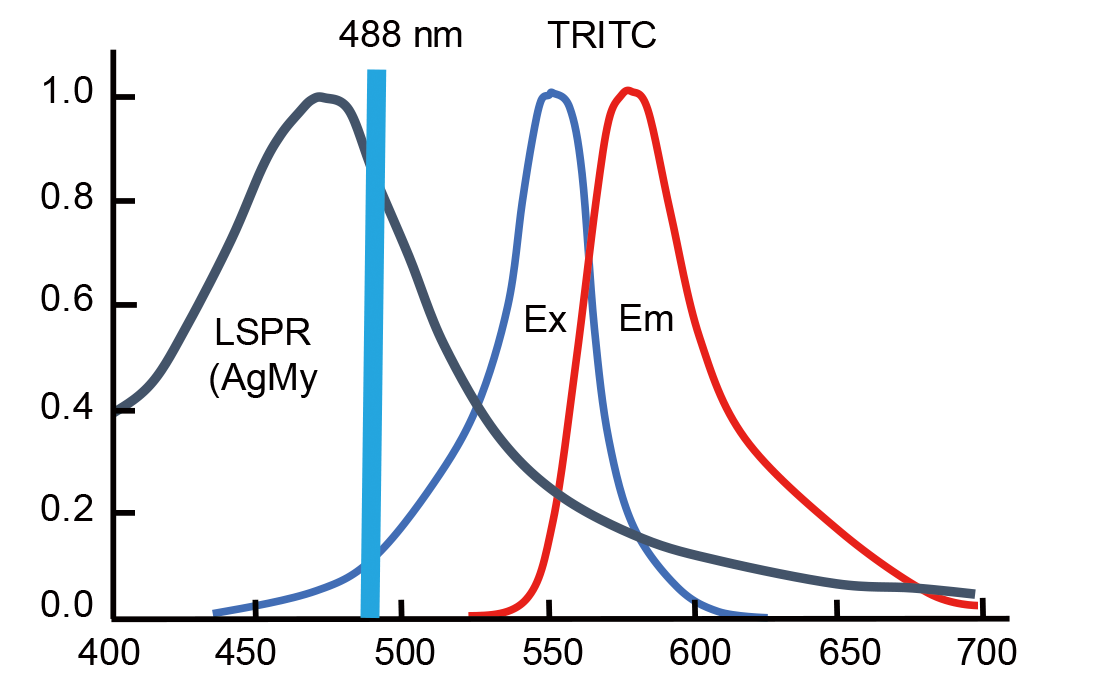

Supplement: S2 Fig — (TIF) [file pone.0189708.s002.tif]

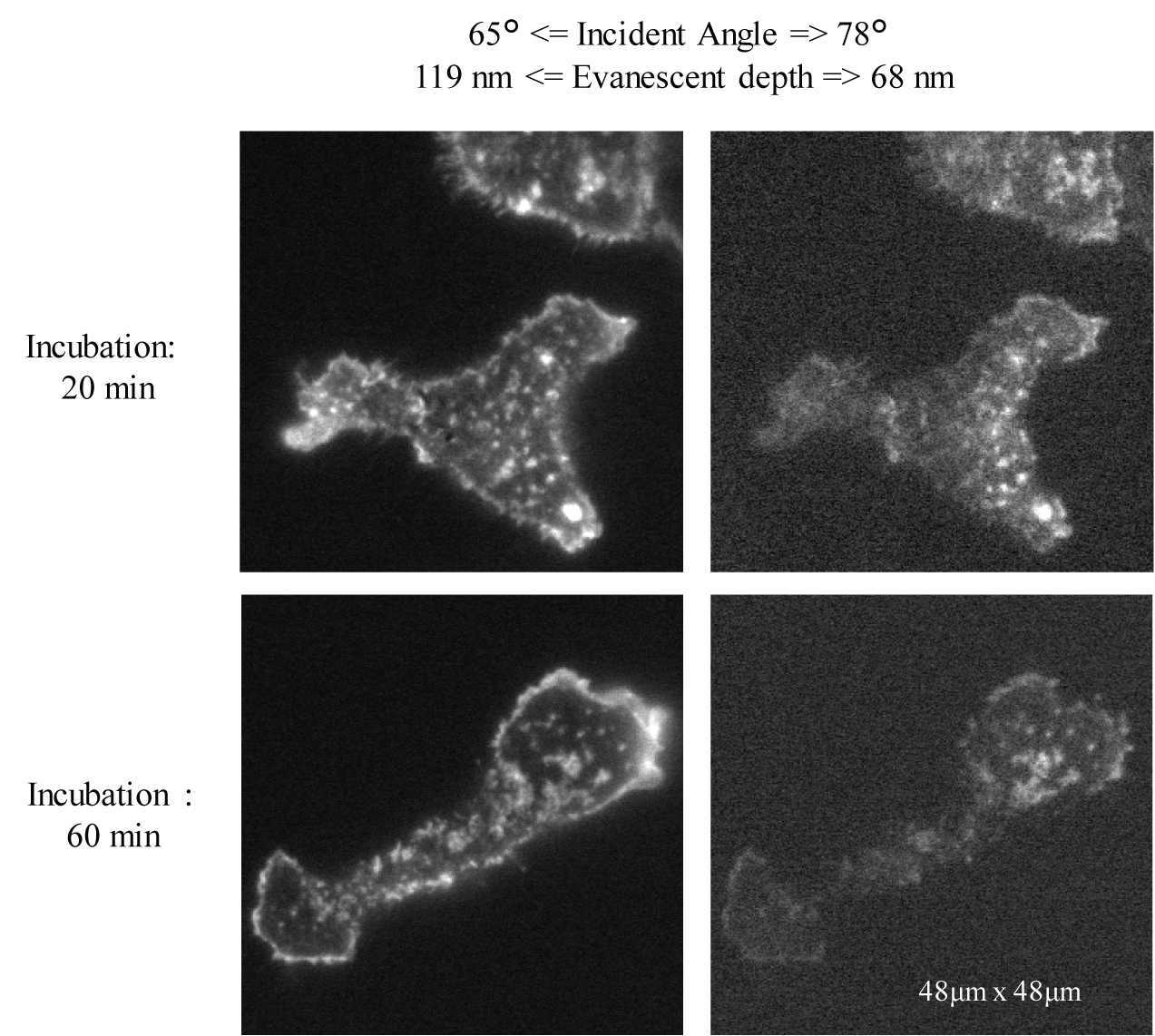

Supplement: S3 Fig — The incubation time for the cell adhesion was 20 min and 60 min. The incubation time for the cell adhesion was 20 min. The incident light was 591 nm in wavelength, and the intensity was 5 mW. The incident angle was 65° (left) and 78° (right). The exposure time was 500 msec. (TIF) [file pone.0189708.s003.tif]

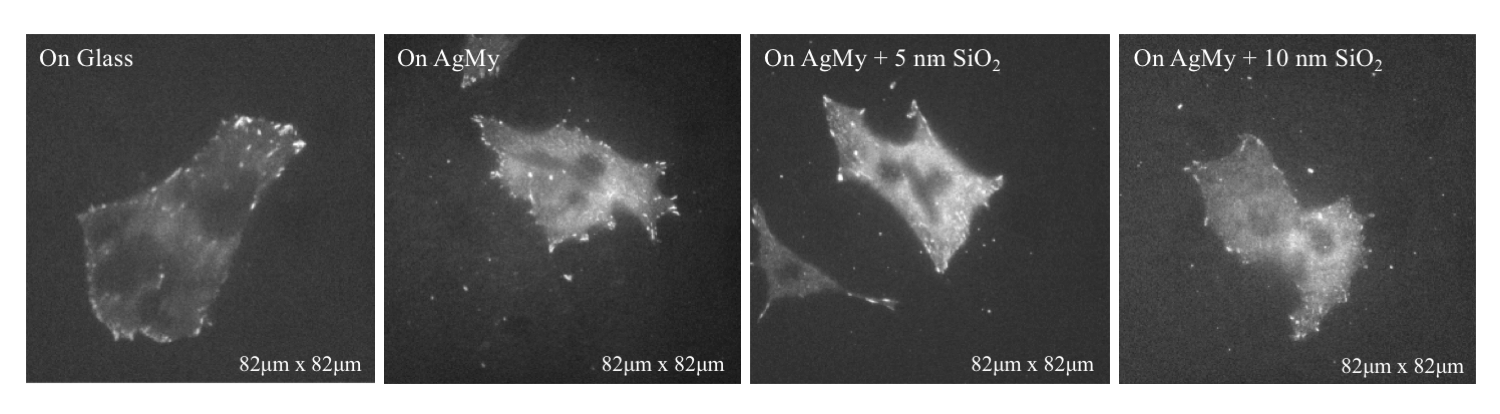

Supplement: S4 Fig — The images were taken by a super-resolution digital CMOS camera (65 nm/pixel). The incident light was 488 nm in wavelength, and the intensity was 7 mW. The incident angle was 62°. The exposure time was 300 msec. (TIF) [file pone.0189708.s004.tif]

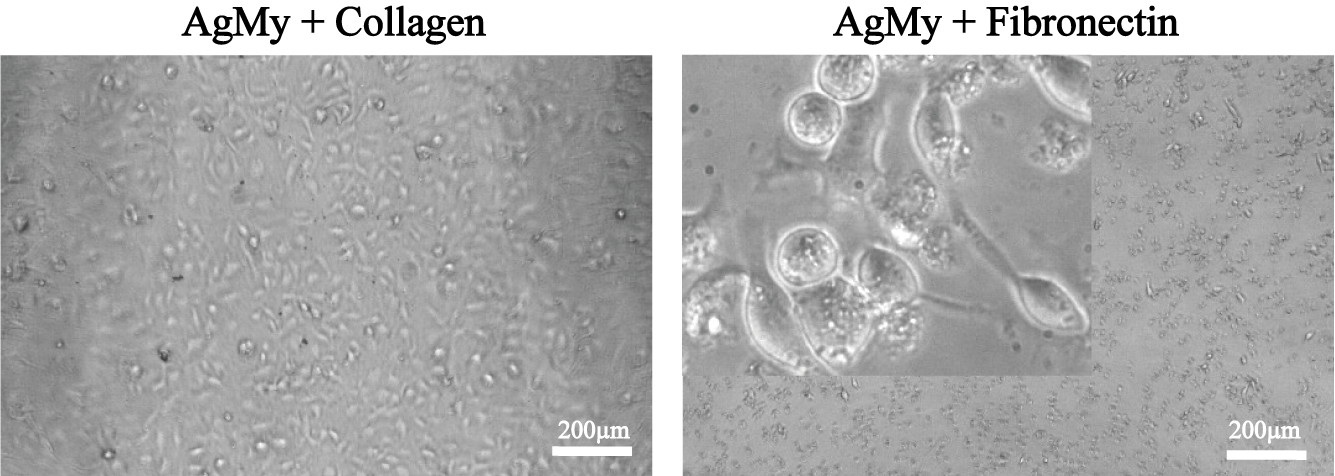

Supplement: S5 Fig — The collagen or fibronectin were spin-coated on top of AgMy sheet. On fibronectin, some cells showed still apoptotic morphology (see the magnified image), which may be due to the inhomogeneous coating with fibronectin in spin-coating. (TIF) [file pone.0189708.s005.tif]
